# Comparative transcriptome analysis of heat-induced domesticated zebrafish during gonadal differentiation

**DOI:** 10.1186/s12863-022-01058-6

**Published:** 2022-05-31

**Authors:** Chenchen Wang, Xuhuai Chen, Yu Dai, Yifei Zhang, Yuandong Sun, Xiaojuan Cui

**Affiliations:** 1 School of Life and Health Science, Hunan University of Science and Technology, Xiangtan, 411201 Hunan China; 2 Hunan Key Laboratory of Economic Crops Genetic Improvement and Integrated Utilization, Hunan University of Science and Technology, Xiangtan, 411201 Hunan China

**Keywords:** Heat stress, Gonad differentiation, FA pathway, Transcriptome, Zebrafish

## Abstract

**Background:**

The influence of environmental factors, especially temperature, on sex ratio is of great significance to elucidate the mechanism of sex determination. However, the molecular mechanisms by which temperature affects sex determination remains unclear, although a few candidate genes have been found to play a role in the process. In this study, we conducted transcriptome analysis of the effects induced by high temperature on zebrafish during gonad differentiation period.

**Results:**

Totals of 1171, 1022 and 2921 differentially expressed genes (DEGs) between high temperature and normal temperature were identified at 35, 45 and 60 days post-fertilization (dpf) respectively, revealing that heat shock proteins (HSPs) and DNA methyltransferases (DNMTs) were involved in the heat-exposed sex reversal. The Gene Ontology (GO) terms and the Kyoto Encyclopedia of Genes and Genomes (KEGG) pathway that were enriched in individuals after heat treatment included Fanconi anemia (FA) pathway, cell cycle, oocyte meiosis and homologous recombination.

**Conclusions:**

Our study provides the results of comparative transcriptome analyses between high temperature and normal temperature, and reveals that the molecular mechanism of heat-induced masculinization in zebrafish is strongly related to the expression of HSPs and DNMTs and FA pathway during gonad differentiation.

**Supplementary Information:**

The online version contains supplementary material available at 10.1186/s12863-022-01058-6.

## Background

Differentiation at high temperature is one of the most critical issues in developmental biology, and plays important roles in evolution, behavior and gonadal development in animals [1–3]. Fish exist a variety of sex determination strategies, including genetic sex determination (GSD), temperature-dependent sex determination (TSD) and GSD + TE (GSD plus temperature effects) [4, 5]. In species with GSD, sex is determined after fertilization and depends on the genetic constitution of an individual, while in TSD species, gonads remain undifferentiated until exposed to environmental temperature at the sensitive period. Interestingly, in GSD + TE species, primary sex determined by the genetic factors, can be changed by extreme environmental temperature [6–9].

In order to reveal the molecular mechanism underlying GSD + TE, the effects of temperature on sex reversal have been investigated in different species, and the following hypotheses have been proposed. Sex hormone has been suspected to play a critical role in temperature influencing sex determination. The *cyp19a1* gene, also known as “aromatase”, which encodes an aromatase that converts androgens into estrogens, is inhibited after exposed to high temperature, resulting in masculinization of individuals [7, 8]. Another typical biological response to temperature is epigenetic modification. Past studies have reported that temperature-specific patterns of DNA methylation and histone modifications of sexual development genes, such as the histone demethylase KDM6B in red-eared slider turtles that regulates transcriptomic of the male sex-determining gene *dmrt1* and leads to sex reverse [4, 9–11]. Findings in Nile tilapia and American alligator suggested that the heat shock proteins also play an important role in the influence of temperature on sex determination [12, 13].

Zebrafish is a small freshwater teleost that has become an important model to study developmental biology, environmental toxicology and medicine in vertebrates. Zebrafish, all individuals develop a “juvenile ovary” prior to the final differentiation on mature ovaries or testes. Ovaries start their differentiation before at 14 dpf and are fully differentiated at 60 dpf [14, 15], on the other hand, testes begin to differentiate at 30 dpf and are fully differentiated about 60 dpf. What’s more, coexistence of degenerating diplotene oocytes and spermatogonia was evident at 26 dpf, and this structure finally transformed into testes at 34 dpf [14]. All males go through the “juvenile ovary-to-testes” transformation process, during which large numbers of apoptotic oocytes have been observed and were thought to play a crucial role in gonadal development [16, 17]. Several signaling pathways have been reported to be involved in the transformation, such as Tp53-apoptosis, NF-κB and canonical Wnt [18–20].

Zebrafish lost sex determining genes during laboratory domestication, and the current studies did not detect heteromorphic sex chromosomes [21, 22]. In the latest years, several molecular studies support that zebrafish has a polygenic sex determination [23–25]. The effects of elevated temperature have been examined in zebrafish [26–28]. In agreement with the general temperature responsive pattern in fish, elevated temperatures result in a higher proportion of males. However, most of the previous experiments have focuses on *cyp19a1*, *dmrt1*, and other genes involved in sex determination or differentiation in zebrafish are known, and little attention has been paid to other genes, such as *dnmt1,* which is involved in DNA methylation modification [26–28]. In addition, the time-course transcriptome analysis of GSD + TE process is minimal by far [28].

Thus, zebrafish provide an excellent opportunity to investigate the influences of temperature on sex determination. In this study, we combined RNA-seq and qPCR approaches to characterize the sex ratio response to temperature in this model, and reveal the genes network responsible for heat-induced masculinization.

## Results

### Effect of rearing temperature on phenotypic sex ratios in zebrafish

**Fig. 1:**
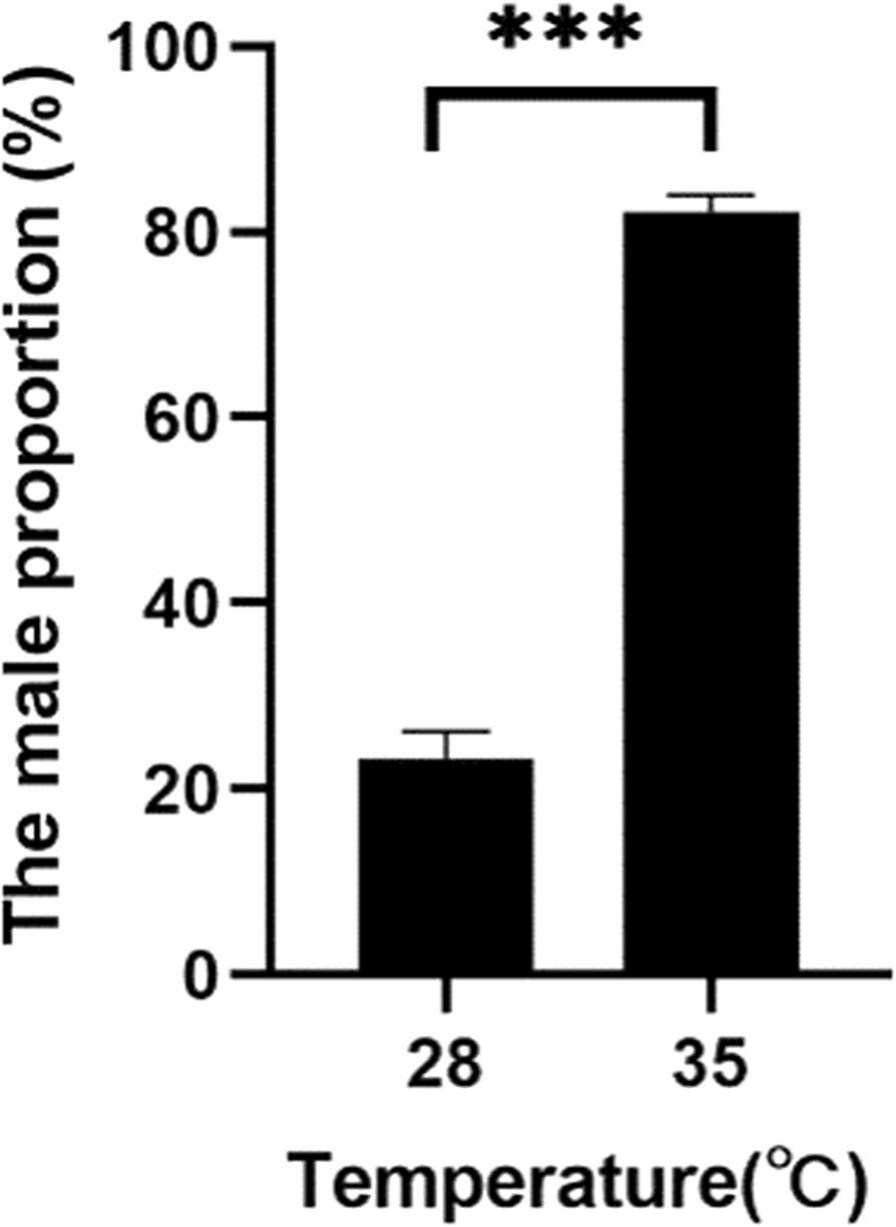
Early treatment (25–35 dpf) with high temperature (35 °C) increased the proportion of males. The mean ± SEM of three biological replicates for control (28 °C; *n* = 95) and treated group (35 °C; *n* = 104) is shown. Statistically significant differences were found between the two sex ratios (***P < 0.001; T-test)

First, we conducted experiments to determine whether high temperature affected individual masculinization. The offspring of 21 adult fish from the same family was obtained to carried out heat treatment experiment during temperature sensitive period (25–35 dpf). The data shown that the male ratio of the normal temperature group was 23.17 ± 3.05% (*n* = 95) (Fig. 1). And the average male ratio of the temperature-treated group (35 °C, 25–35 dpf) was 82.16 ± 1.79%(*n* = 104), a significant increase in the masculinization rate was observed compared with the normal temperature group (p<0.001) (Fig. 1).

### Phenotypic identification and analysis of adult zebrafish gonads

**Fig. 2:**
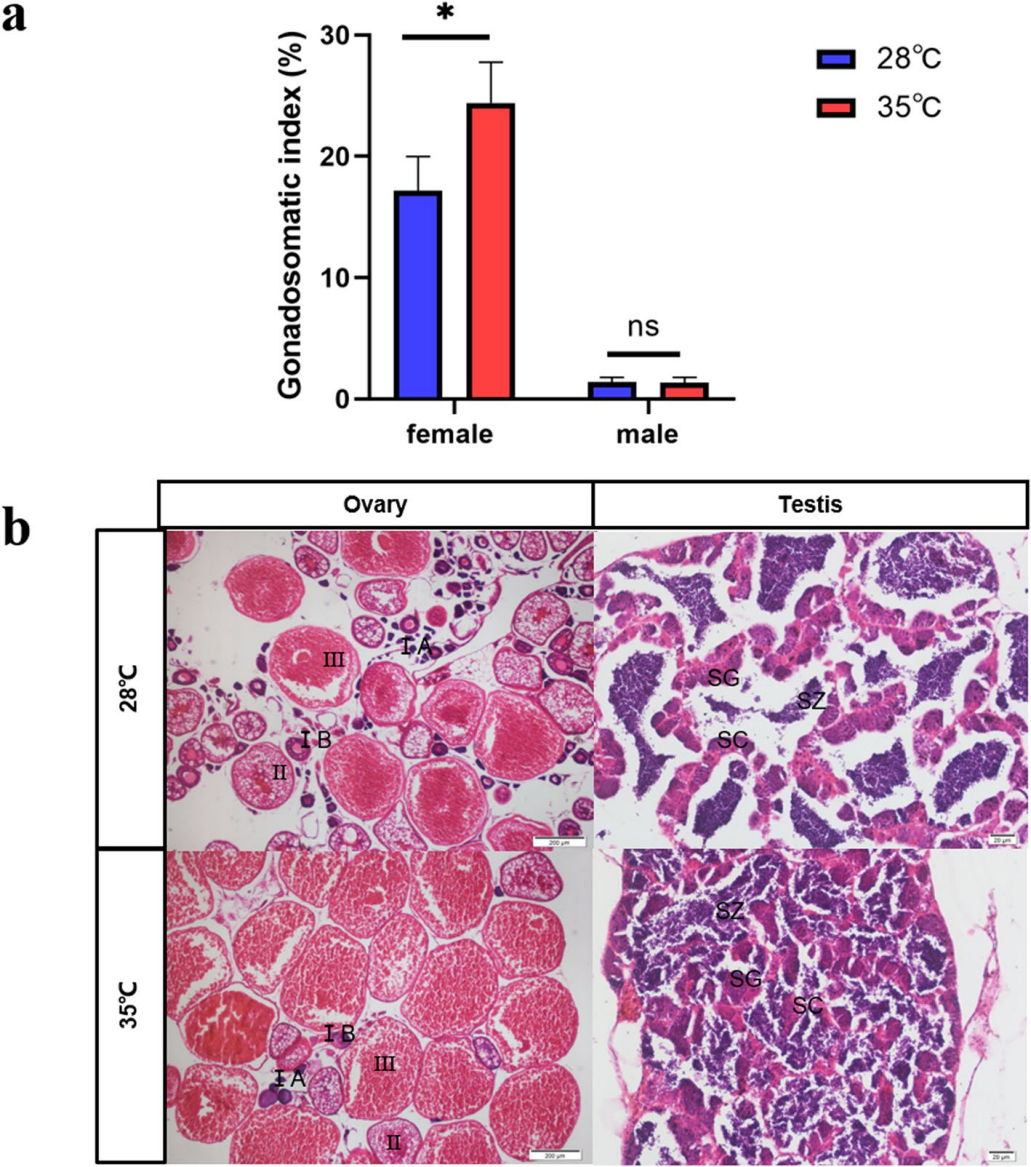
Phenotypic identification and analysis of temperature-treated fish. (**a**) the comparative analysis of GSI between high temperature (35 °C) and normal temperature (28 °C) (T-test, *P < 0.05). (**b**) Histological analysis of gonad tissue. Stage IA and stage IB, primary growth stage; stage II, cortial alveolus stage; stage III, vitellogenesis stage; SC, spermatocytes; SG, spermatogonia; SZ, spermatozoa

To explore the heat treatment effect on gonadal development, the gonadosomatic index (GSI) and histological analysis were performed for adult fish at 90 dpf. The average GSI of adult female in high temperature group was 24.42% and that in control temperature group was 17.19%, indicating a significant difference (*P* < 0.05) (Fig. 2a). The GSI analysis of males compared between high and normal temperature-treated also carried out, but there was not statistical difference (Fig. 2a). With regard to the terms of developmental stages of oocytes in zebrafish, there were appeared a series of oocyte maturation stages, including stage IA, stage IB, stage II and stage III in the ovaries of the control group. Among the 20 females from thermal treatment group, 16 of them were observed with several stage IA, stage IB and few stage II oocytes in their ovaries (Fig. 2b).

### Transcriptomic analysis between fish exposed to high and normal temperature

To investigate the effects of temperature during gonad differentiation, RNA-seq on the BGISEQ-500 platform was used to sequence the cDNA library from the whole body of zebrafish at 25, 35, 45 and 60 dpf respectively. A mean of 45.51 M filtered clean reads were obtained from each sample. 82.18% of which mapped to the *Danio rerio* genome (see Additional file 1). Traditionally, qRT-PCR is used to validate the expression levels quantified by high throughput technology RNA-seq. Therefore, 20 candidate genes associated with cell cycle, oocyte meiosis and homologous recombination were selected from hundreds of differently expressed transcripts between the high and normal temperature treated groups (see Additional file 2 and Additional file 3). Then, the Person’s correlation coefficient indicated that the expression levels were found to be highly reliable for genes that are determined to be significantly differentially expressed by RNA-seq (r = 0.73, *p*-value< 0.0001) (see Additional file 5).

**Fig. 3:**
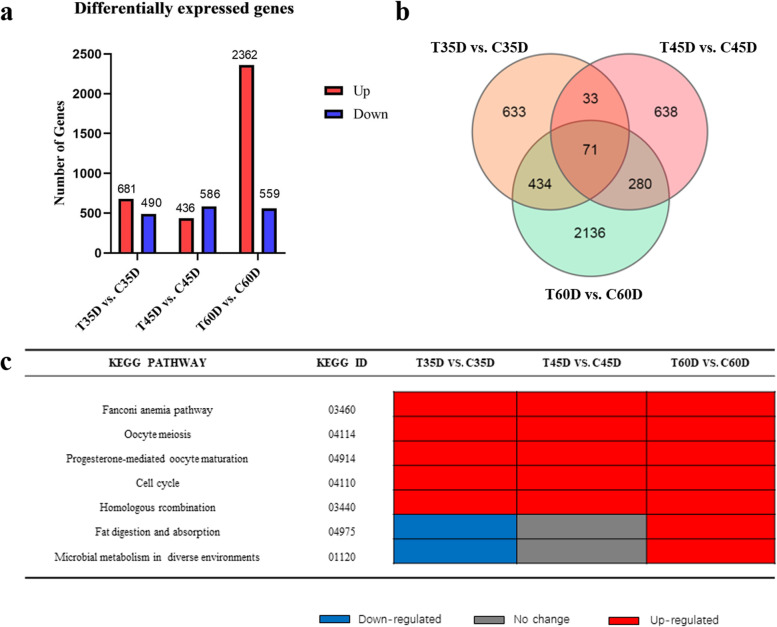
Analysis of DEGs between high temperature and normal temperature treatments at 35dpf, 45dpf and 60dpf. Number of DEGs identified from each comparison groups (**a**), shown by Venn diagram (**b**), and list of pathways [29, 30] involved in the gonad development in juvenile fish (**c**)

To explore differences in gene expression between high temperature and normal temperature treatments during gonadal differentiation, we conducted comparative analysis of different expression genes on the three transcriptomic groups, T35D vs. C35D, T45D vs. C45D and T60D vs. C60D respectively (Fig. 3a). Venn diagram was shown (Fig. 3a). To determine the biological function of DEGs during gonadal differentiation after heat-induced, GO classification and KEGG pathway enrichment analysis were performed. (Additional file 6).

Comparing T35D and C35D, there were 1171 DEGs, including 681 up-regulated and 490 down-regulated genes (Fig. 3a). GO analysis of DEGs between T35D and C35D was conducted, and the results revealed that the subclasses double−strand break repair, DNA recombination (in biological process category), DNA polymerase activity, nuclease activity (in molecular function category), chromosomal region, replication fork (in cellular component category) were the aspects significantly regulated by heat-induced (Additional file 6a). These results suggest that DNA damage repair and DNA replication were mainly involved in heat-induced of zebrafish. The results of KEGG enrichment analysis revealed that top 20 pathways were significantly enriched(q < 0.05), including FA pathway, cell cycle, oocyte meiosis, homologous recombination and progesterone-mediated oocyte maturation, which were up-regulated (Fig. 3c and Additional file 6d). Especially in FA pathway, 13 genes including *fancf*, *fancg*, *fanci* and *fanco* were up-regulated in heat-treated fish compared to normal control fish (Fig. 3c and Additional file 3). In addition, the expression level of genes coding DNA methyltransferase family members (*dnmt1*, *dnmt3bb.2*) and heat shock proteins (*hsp90a*, *hsp70l*, and *hspbp1*) were also significant upregulated in high-temperature group except *hsp4l* gene (Additional file 3 and Additional file 5).

Genes expression changes in individuals maintained at high temperature conditions were identified by comparing T45D and C45D. Total of 1022 DEGs, including 436 up-regulated and 586 down-regulated genes (Fig. 3a). The GO analysis results showed that the enriched GO terms were visual perception, synaptic vesicle localization and transport (in biological process category), structural constituent of eye lens, aminoacyl-tRNA ligase activity (in molecular function category), photoreceptor cell cilium, transmembrane transporter complex (in cellular component category) (Additional file 6b). These results suggest that increased temperature affects light perception and synaptic vesicle. In addition, there were top 20 enriched KEGG pathways(q < 0.05), including synaptic vesicle cycle, glutamatergic synapse, fatty acid elongation and dopaminergic synapse (Additional file 6e).

Genes that were differentially expressed in high-temperature group and control group after sex differentiation were evaluated by identifying DEGs in T60D vs C60D. There were 2921 DEGs, including 2362 up-regulated and 559 down-regulated genes (Fig. 3a). The GO analysis results of significantly terms were double−strand break repair, ribosome biogenesis (in biological process category), methyltransferase activity, S-adenosylmethionine-dependent methyltransferase activity (in molecular function category), replication fork, chromosomal region (in cellular component category) (Additional file 6c). From the above results, it is suggested that ribosome biogenesis, transferase activity and DNA replication process were mainly involved in heat-induced of zebrafish. KEGG enrichment analysis identified top 20 pathways(q < 0.05), including metabolic pathway, phototransduction and steroid biosynthesis (Additional file 6f).

## Discussion

Temperature represents one of abiotic factors essential for the development and growth of fish species [31, 32]. The sex reversal effects of high temperature were carried out in zebrafish or other fish species. However, previous researches mainly focused on early embryonic period or different expression genes related to sex differentiation in adult [7, 26–28, 33]. While, we performed transcriptome gene expression analysis during the sex differentiation period in zebrafish to investigate the time-course effects of temperature on sex determination.

Numerous studies have shown that DNA damage repair system, heat shock proteins and other factors may effectively respond to heat stress, also known as heat shock response (HSR), which is a defensive adaptive response characterized by changes in gene expression under environmental stress [34–37]. In our study, GO enrichment analysis results at 35dpf and 60dpf showed that processes such as double-strand break repair, DNA polymerase activity and nuclease activity were significantly regulated by heat induction, suggesting that the DNA damage repair system contributes to HSR. In addition, most HSPs are rapidly produced via heat shock factor (HSF)-regulated gene expression in stressed cells during HSR [37, 38]. However, it is worth mentioning that HSP70 and HSP47 were induced by thermal stress, whereas HSP90A, HSP90B and HSF1 were expressed constitutively and not induced by heat stress in zebrafish [31, 39–41]. Interestingly, we observed that exposure to thermal increased the transcript levels of *hsp90a*, *hsp70l* and *hspbp1*, and decreased *hsp4l*, suggesting that HSPs are also involved in other biological processes.

Previous evidence suggests that pathways such as HSR, FA pathway and cytochrome P450 are critical in gonadal differentiation [7, 8, 39, 42–44]. First of all, HSR-related factors such as HSP90A, HSPBP1, HSP4l and HSF5 are involved in spermatogenesis and fertilization in mammals [45–48]. Particularly, HSP90 is a major molecular chaperones that even mediated estrogen receptor α (ERα) methylation and repressed the ERα activity [49]. Our data support the works in Nile tilapia and American alligator that the HSPs may play multiple roles in TSD, but further research is needed [12, 13].

Furthermore, several gonadal development-related pathways were upregulated in heat-exposed zebrafish compared to normal group, including FA pathway, oocyte meiosis, progesterone-mediated oocyte maturation, cell cycle and homologous recombination. We focused on the FA pathway and discovered that the expression of *uhrf1*, *fancf* and other 11 genes were raised in heat-temperature compared to normal temperature. Surprisingly, we detected a significant increase in the expression level of FA signaling pathway after heat stress, which is reported for the first time. What’s more, previous studies have shown that there is crosstalk between FA pathway and cell cycle and homologous recombination pathway, even playing an important role in gonads sterility and sex-reversion [42–44]. Therefore, we hypothesize that the FA signaling pathway may play an important role in heat-exposed masculinization in collaboration with other signaling pathways.

In many organisms, genome-wide methylation level in testis (or ovaries) are significantly higher in the hyperthermia group than in the normal group, and epigenetic remodeling is widely believed to play a crucial role in heat-induced masculinization [4, 5, 9, 10, 50]. However, these works focused on differentially expressed genes in sexually mature individuals, and no significant difference was detected in *dnmts* gene expression. Nevertheless, in this study, we tracked DEGs during gonadal differentiation and found the expression level of the DNA methyltransferase genes *dnmt1* and *dnmt3bb.2* were up-regulated under heat temperature at 35, 45 and 60 dpf, consistent with the results of Dorts et al [51] Strangely, the results of Ribas et al. showed that *dnmt3bb.2* was downregulated after high temperature treatment [28], opposite to our results, which may be related to acute heat treatment or chronic heat treatment. At present, DNMT3A and DNMT1 have been found the effect on fertility by programming the methylation patterns of germ cells via cell cycle process in mammal [52–54]. So, what’s the role of *dnmts* in heat-induced masculinization in zebrafish? Is there an (direct or indirect) interaction between DNA methyltransferase and histone methylation in high-temperature sex reversal? Whole Genome Bisulfite Sequencing approaches combined with classical reverse genetic methods are needed to reveal these questions.

## Conclusions

In our study, we present the RNA-seq analysis to learn the mechanism of heat-induced masculinization in zebrafish. Our data demonstrated elevated temperature during the temperature sensitive period leads to an increase in male ratio in domesticated zebrafish, supporting a GSD + TE sex determination. We also show that normal ovarian development is somewhat inhibited in individuals exposed to high temperature. Strikingly, we detected DNA methyltransferase genes (*dnmt1* and *dnmt3bb.2*) and FA pathway were significantly upregulated after heat treated for the first time. These results provide insights into heat-induced sex reversal and help us further understanding the mechanisms of vertebrata sex determination.

## Methods

### Fish and ethics statement

The AB strain were maintained in a glass aquarium with a circulating water system at 28 ± 0.5 °C, with photoperiods of 14 L-10D. All procedures were approved by the Animal Care and Use Committee of Hunan University of Science and Technology. Fish were maintained and conducted in compliance with the ARRIVE guideline [27].

### Embryo collection and thermal treatment

In order to avoid the influence of different families on the sex ratio, 21 fish (14 females and 7 males) from the same family mating took place spontaneously in spawning tanks. Fertilized eggs were collected and separated in Petri dishes and maintained at 28 ± 0.5 °C. Eggs hatch at 3 dpf and total 1200 larvae were divided into 2 groups as follows. The control group was kept at 28 °C, whereas the thermal treatment group was exposed to 35 °C for 10 days during 25–35 dpf. And each group had three biological replicates. In order to avoid the effect of high density on gonad differentiation, the number of fish in each 5-liter tank was 50–55. After the treatment, fish were returned at 28 °C until the fish reached to sexual maturity (90 dpf).

### The GSI and histological analysis in fish

Dissected gonads from 20 adult individuals of each group at 90 dpf were analyzed. First, their gonads were removed and weighed immediately. The GSI was calculated as the ratio of gonad (W_g_) to the total fish weight (W), expressed as a percentage, GSI = [(W_g_ /W) × 100]. Then, gonads were fixed in 4% paraformaldehyde for 24 h at room temperature, washed in phosphate buffered saline, dehydrated in an ascending gradient of ethanol concentrations, cleared in xylene and embedded. 14 μm thick sections were cut and flatted on glass slides, stained with haematoxylin and eosin and sealed with neutral resin. Sections were examined with a light microscope and images were captured.

### Total RNA extraction

5–15 fish were collected at 25, 35, 45 and 60 dpf. Three independent biological replicates were processed at each time node and temperature. Thus, 21 samples labeled as C25D-1 ~ 3, C35D-1 ~ 3, C45D-1 ~ 3, C60D-1 ~ 3, T35D-1 ~ 3, T45D-1 ~ 3, and T60D-1 ~ 3 were acquired. Total RNA for each sample was extracted from the whole body of fish using Trizol (Invitrogen, USA) according to manual instruction. About 60 mg of tissues were ground into powder by liquid nitrogen in a 2 mL tube, followed by being homogenized for 2 minutes and rested horizontally for 5 minutes. The mix was centrifuged for 5 minutes at 12,000×g at 4 °C, then the supernatant was transferred into a new EP tube with 0.3 mL chloroform/isoamyl alcohol (24:1). The mix was shacked vigorously for 15 s, and then centrifuged at 12,000×g for 10 minutes at 4 °C. After centrifugation, the upper aqueous phase where RNA remained was transferred into a new tube with equal volume of supernatant of isopropyl alcohol, then centrifuged at 13600 rpm for 20 minutes at 4 °C. After deserting the supernatant, the RNA pellet was washed twice with 1 mL 75% ethanol, then the mix was centrifuged at 13600 rpm for 3 minutes at 4 °C to collect residual ethanol, followed by the pellet air dry for 5–10 minutes in the biosafety cabinet. Finally, 25–100 μL of DEPC-treated water was added to dissolve the RNA. Subsequently, total RNA was qualified using a Nano Drop and Agilent 2100 bioanalyzer (Thermo Fisher Scientific, USA).

### mRNA library construction

Oligo (dT)-attached magnetic beads were used to purified mRNA. Purified mRNA was fragmented into small pieces with fragment buffer at appropriate temperature. Then first-strand cDNA was generated using random hexamer-primed reverse transcription, followed by a second-strand cDNA synthesis. Afterwards, A-Tailing Mix and RNA Index Adapters were added by incubating to end repair. The cDNA fragments obtained from previous step were amplified by PCR, and products were purified by Ampure XP Beads, then dissolved in EB solution. The product was validated on the Agilent Technologies 2100 bioanalyzer for quality control. The double stranded PCR products from previous step were heated denatured and circularized by the splint oligo sequence to get the final library. The single strand circle DNA (ssCir DNA) was formatted as the final library. The final library was amplified with phi29 to make DNA nanoball (DNB) which had more than 300 copies of one molecular, DNBs were loaded into the patterned nanoarray and single end 50 bases reads were generated on BGISEQ-500 platform (BGI-Shenzhen, China).

### RNA-sequencing data analysis

The sequencing data was filtered with SOAPnuke (v1.5.2) [55] by removing reads containing sequencing adapter; removing reads whose low-quality base ratio (base quality less than or equal to 5) is more than 20%; removing reads whose unknown base (‘N’ base) ratio is more than 5%, afterwards clean reads were obtained and stored in FASTQ format. The clean reads were mapped to the reference genome using HISAT2(v2.0.4) pipeline [56]. Bowtie2 (v2.2.5) [57] was applied to align the clean reads to the reference coding gene set (GCF_000002035.6_GRCz11), then expression level of gene was calculated by RSEM (v1.2.12) [58]. Essentially, differential expression analysis was performed using the DESeq2(v1.4.5) [59] with Q value ≤0.05. To take insight to the change of phenotype, GO (http://www.geneontology.org/) and KEGG (https://www.kegg.jp/) enrichment analysis of annotated DEGs were performed by Phyper (https://en.wikipedia.org/wiki/ Hypergeometric_distribution) based on Hypergeometric test. The significant level of terms and pathways were corrected by Q value with a rigorous threshold (Q value≤0.05) by Bonferroni [60].

### Validation of transcriptome data by real-time PCR

To verify accuracy of the transcriptome data, qRT-PCR analyses were performed using samples from 35, 45 and 60 dpf. Twenty candidate genes with key functions in cell cycle, oocyte meiosis and homologous recombination were randomly selected for qRT-PCR analysis. Total RNAs were reverse transcribed into cDNAs with the Primescript™ RT reagent kit (Takara, Japan). The PCR reaction system comprised the following ingredients: 10 μl 2 × SYBR Green PCR master Mix (Biorad, China), 1 μl of each primer, 2 μl of template cDNA, and 6 μl of RNase-free ddH2O. The cycling program was carried out under the following protocol: 95 °C for 1 min, 40 cycles with each cycle for 15 s at 95 °C, 15 s at 60 °C, and 30 s at 72 °C, before finally ramping from 65 to 95 °C at 0.5 °C per 5 s to generate a melting curve demonstrated no dimer primers, nor unwanted additional PCR products. The zebrafish *eef1a1l1* and *β2m* genes were selected as the control genes. Each qRT-PCR experiments was repeated three times. The relative expression levels of 20 genes in different samples were calculated using the 2^-ΔΔCT^ method among three biological replicates. The primers employed were listed in the Additional file 4.

### Statistical analyses

GraphPad Prism 8 was used to analyze all statistical data and make the graphs. Data are presented as the mean ± SEM. Differences between experimental group were made by independent sample T-test. Then the Pearson’s correlation analysis for the data of RNA-seq and qRT-PCR was performed. The statistical significance was declared at *P* < 0.05, *P* < 0.01 or *P* < 0.001.

## Supplementary Information

**Additional file 1 **Summary statistics of the transcriptome sequencing of *D. rerio*.

**Additional file 2.** Type of heat effect on gene in juveniles at 35, 45 and 60 dpf.

**Additional file 3.** RNA-Seq and qRT-PCR validation results.

**Additional file 4.** Gene symbols, Refseq IDs and primer sequences for all genes used in qPCR.

**Additional file 5 Fig. S1.** The person’s correlation coefficient between RNA-Seq and qRT-PCR.

**Additional file 6 Fig. S2.** Enriched GO terms and KEGG pathways.

## Data Availability

The complete clean data have been uploaded to NCBI’s Gene Expression Omnibus (GEO) with accession number GSE201748 (https://www.ncbi.nlm.nih.gov/geo/).

## Abbreviations

DEGs: Differentially expressed genes
DPF: Days post-fertilization
HSPs: Heat shock proteins
DNMTs: DNA methyltransferases
GO: Gene Ontology
KEGG: Kyoto Encyclopedia of Genes and Genomes
FA: Fanconi anemia
GSD: Genetic sex determination
TSD: Temperature-dependent sex determination
GSD + TE: GSD plus temperature effects
GSI: Gonadosomatic index
HSR: Heat shock response
HSF: Heat shock factor
ERα: Estrogen receptor α
