# Supplementary material for: Comparative transcriptome analysis of heat-induced domesticated zebrafish during gonadal differentiation
Source: BMC Genom Data. 2022 May 31;23:39. doi: 10.1186/s12863-022-01058-6 (PMC9158171; doi:10.1186/s12863-022-01058-6)
Supplement: Supplementary file 5 — Additional file 5 Fig. S1. The person’s correlation coefficient between RNA-Seq and qRT-PCR. [file 12863_2022_1058_MOESM5_ESM.docx]

Additional file 5. The person's correlation coefficient between RNA-Seq and qRT-PCR.


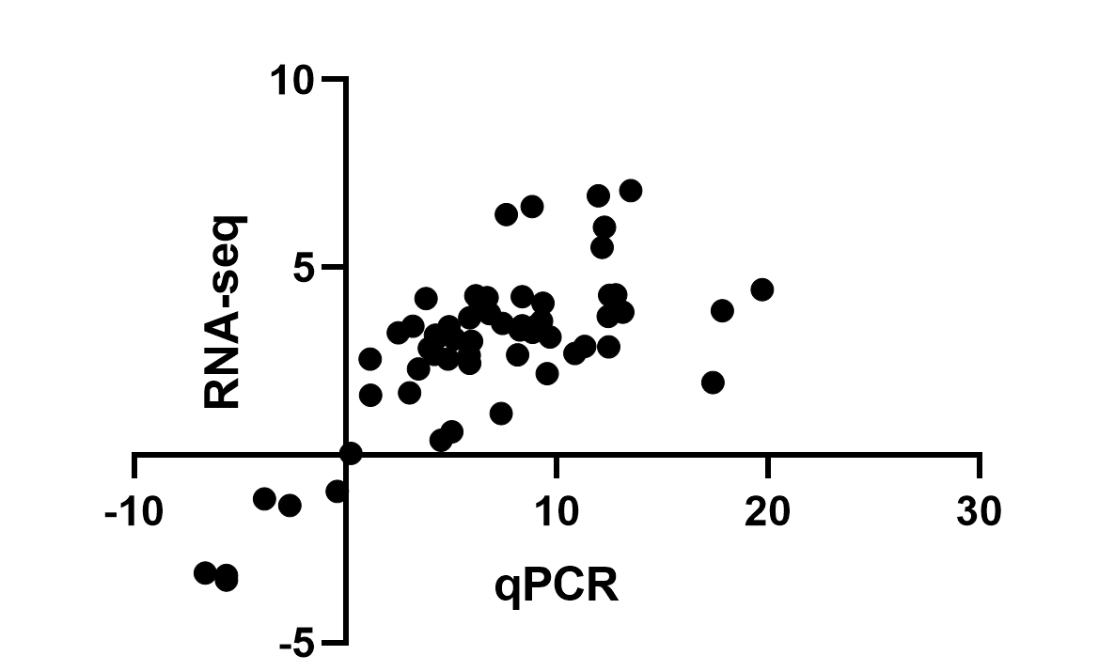


Additional file 5. The person's correlation coefficient between RNA-Seq and qRT-PCR. Pearson correlation coefficients were used to calculate the expression correlation of 20 genes at 35, 45, and 60 dpf for RNA-seq and qRT-PCR (r=0.73, p-value<0.0001).
