# Supplementary material for: Comparative transcriptome analysis of heat-induced domesticated zebrafish during gonadal differentiation
Source: BMC Genom Data. 2022 May 31;23:39. doi: 10.1186/s12863-022-01058-6 (PMC9158171; doi:10.1186/s12863-022-01058-6)
Supplement: Supplementary file 6 — Additional file 6 Fig. S2. Enriched GO terms and KEGG pathways. [file 12863_2022_1058_MOESM6_ESM.docx]

Additional file 6. Enriched GO terms and KEGG pathways.


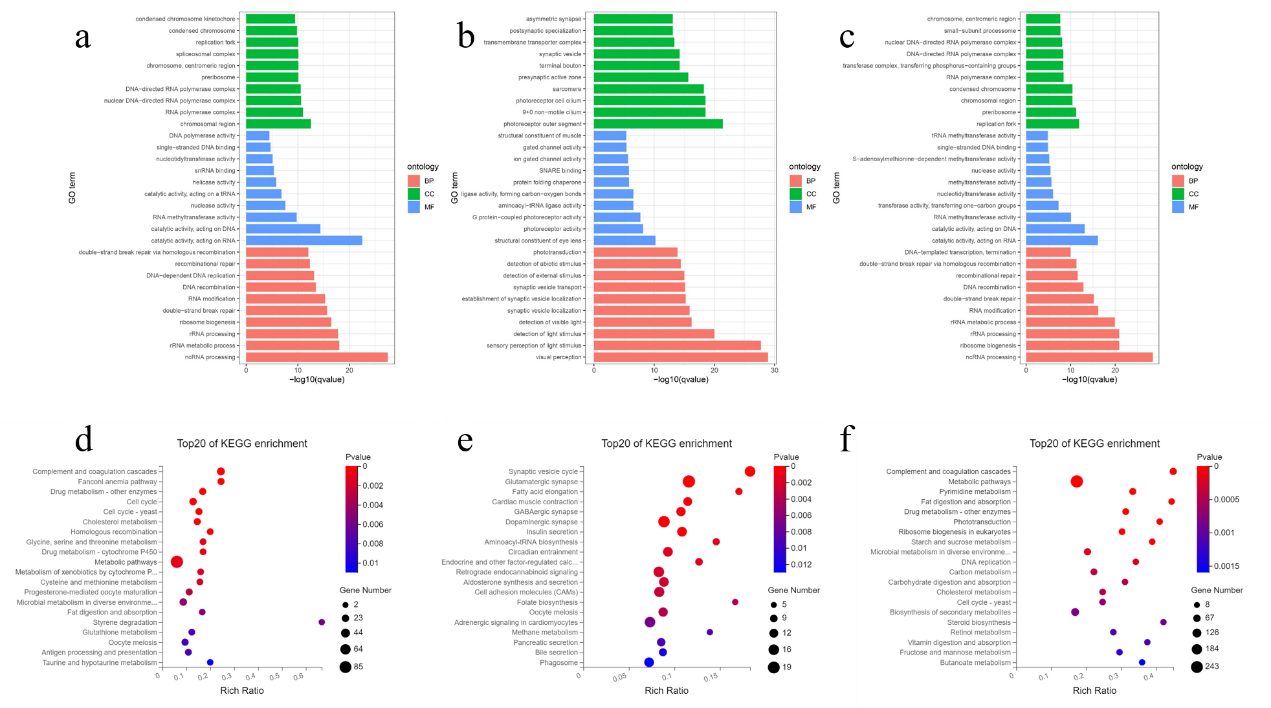


Additional file 6. Enriched GO terms and KEGG pathways. Top 10 statistics of GO enrichment analysis of DEGs in zebrafish upon heat-induced. GO analysis of DEGs between high temperature and normal temperature treatments at 35dpf (a), 45dpf (b) and 60dpf (c). The DEGs caused by high temperature matched various GO categories, including biological process (BP), cellular component (CC), and molecular function (MF). The x-axis indicates the degree of enrichment and the y-axis indicates the GO terms. Top 20 statistics of pathway enrichment based on the DEGs. Bubble diagram of KEGG pathway enrichment statistics after heat-induced in zebrafish at 35dpf (d), 45dpf (e) and 60dpf (f). Rich Radio is the ratio of differentially expressed gene numbers noted in this pathway term to all gene numbers noted in this pathway term, greater Rich Radio means greater intensiveness, and the less P-value means greater intensiveness. The size of the bubbles indicates the number of DEGs enriched in each pathway.
